# Correlation between blood biomarkers and post-stroke cognitive impairment

**DOI:** 10.3389/fnagi.2025.1736338

**Published:** 2026-01-13

**Authors:** Xianjun Liu, Zhaoyang Lv, Zhihong Shi, Feng Liu, Hao Wu, Shuai Liu, Yong Ji

**Affiliations:** 1 Department of Neurology, Huanhu Hospital Affiliated to Tianjin Medical University, Tianjin, China; 2 Tianjin Key Laboratory of Cerebrovascular and Neurodegenerative Diseases, Tianjin Dementia Institute, Tianjin Huanhu Hospital, Tianjin, China; 3 Department of Neurology, The Central Hospital of Yongzhou, Yongzhou, Hunan, China

**Keywords:** blood biomarkers, correlation, GFAP, NFL, post-stroke cognitive impairment, prediction

## Abstract

**Background:**

Post-stroke cognitive impairment (PSCI) is common and imposes a significant burden upon both families and society. There is limited information on biomarkers for PSCI. This study investigated the correlation between blood biomarkers and post-ischaemic stroke cognitive impairment, to identify potential blood biomarkers and their efficacy in predicting the disorder.

**Methods:**

This prospective study enrolled patients who had experienced their first acute ischaemic stroke between January 2024 and March 2025. Patients underwent blood tests within 24 h of admission, which measured plasma levels of Aβ1-40, Aβ1-42, glial fibrillary acidic protein (GFAP), neurofilament light chain (NFL), p-Tau181, p-Tau217, Aβ42/40, and p-Tau217/Aβ1-42. The cognitive function of the patients was assessed at the three-month follow-up visit using the Montreal Cognitive Assessment (MOCA) scale. Participants were divided into a cognitive impairment group and a cognitively normal group with a MoCA cutoff score of 22.

**Results:**

A total of 128 patients who had experienced a first ischaemic stroke were included in the analysis. At the three-month post-stroke follow-up, 69 patients (53.9%) were allocated to the PSCI group, with 59 patients (46.1%) in the cognitively normal group. After univariate and multivariate logistic regression analyses, plasma GFAP (OR = 1.0027, 95% CI = 1.0002–1.0053, *p* = 0.038) and plasma NFL (OR = 1.0046, 95% CI = 1.0006–1.0086, *p* = 0.025) were identified as independent risk factors for cognitive impairment following ischaemic stroke. Receiver operating characteristic (ROC) curves indicated area under the curve (AUC) values of 0.779 (95% CI = 0.700–0.858, *p* < 0.001) for plasma GFAP and 0.809 (95% CI = 0.733–0.885, *p* < 0.001) for plasma NFL, indicating good predictive performance for both parameters. The AUC for GFAP+NFL was 0.855 (95% CI = 0.792–0.918, *p* < 0.001), indicating superior predictive performance of the GFAP and NFL combination for PSCI post-ischaemic stroke cognitive impairment.

**Conclusion:**

Elevated plasma GFAP and NFL levels are associated with an increased risk of post-ischaemic stroke cognitive impairment. Plasma GFAP and NFL may represent potential biological markers for PSCI. The combination of the two parameters showed superior predictive efficacy for PSCI.

## 1 Introduction

According to the China Stroke Report (Wang et al., 2020), the annual incidence of stroke in China is 246.8 per 100,000 population, with a prevalence rate of 1,114.8 per 100,000 population, representing a considerable burden (GBD 2016 Causes of Death Collaborators, 2017). Approximately one-third of stroke patients develop post-stroke cognitive impairment (PSCI) (El Husseini et al., 2023). PSCI represents a clinical syndrome characterized by cognitive impairment following a stroke event (El Husseini et al., 2023; Lee et al., 2023), emphasizing the causal relationship between the stroke and cognitive dysfunction. Based on previous research (El Husseini et al., 2023; Han et al., 2024), we used assessments of cognitive function to identify PSCI.

Vascular cognitive impairment (VCI) refers to cognitive dysfunction resulting from cerebrovascular disease and its associated risk factors (Rundek et al., 2022). PSCI represents a subtype of VCI and encompasses cognitive impairment following both ischaemic and hemorrhagic strokes. Research indicates that patients with PSCI have significantly poorer quality of life and higher mortality rates than those without cognitive impairment. Consequently, the early prediction, identification, and prompt treatment of patients at risk of PSCI is a focal point in contemporary stroke research and a priority for clinical intervention.

Various studies have investigated potential biomarkers that could improve the prediction and diagnosis of PSCI. These potential biomarkers include genetic markers (such as apolipoprotein E), molecular imaging techniques (such as positron emission tomography-computed tomography [PET-CT]), and blood biomarkers (such as *β*-amyloid and tau protein). The collection of peripheral blood is both simple and non-invasive, compared to obtaining cerebrospinal fluid, and blood biomarkers have thus received the most attention (Jiang et al., 2025; Kim et al., 2022). Previous studies on blood biomarkers for PSCI have focused largely on individual factors, such as tau protein, glial fibrillary acidic protein (GFAP), and neurofilament light chain (NFL). The present study aimed to investigate blood factors that could predict the risk of developing PSCI, specifically examining the association between eight factors, namely, plasma levels of Aβ1-40, Aβ1-42, GFAP, NFL, p-Tau181, p-Tau217, Aβ42/40, and the p-Tau217/Aβ1-42 ratio, and PSCI. The findings provide a reference for the prediction of PSCI development, enabling the timely diagnosis and treatment of affected patients.

## 2 Materials and methods

### 2.1 Study subjects

This study is a cross-sectional investigation. This prospective study enrolled patients who had experienced their first ischaemic stroke and who were admitted to Tianjin Huanhu Hospital between January 2024 and March 2025. Inclusion criteria were: (1) First occurrence of acute ischaemic stroke, with onset within the past 7 days; (2) Aged between 50 and 80; (3) Minimum educational attainment of ≥3 years, with the ability to cooperate in completing assessments such as the MOCA; (4) Provision of written informed consent from the patient, indicating willingness to participate in the study. Exclusion criteria: (1) Pre-existing diagnosis of cognitive impairment, psychiatric disorders, traumatic brain injury, encephalitis, or similar conditions before stroke onset; (2) Severe organ failure associated with a life-threatening critical condition; (3) Severe neurological deficits (such as profound aphasia, severe hemiplegia, or significant visuospatial neglect) preventing completion of cognitive assessment. This study had been registered in the Chinese Clinical Trials Registry (ChiCTR2400080663). The study protocol was approved by the Committee for Medical Research Ethics at Tianjin Huanhu Hospital (ID: 2023-157 and 2024-175).

### 2.2 Measurement of plasma biomarkers

Fasting venous blood samples were collected in EDTA anticoagulant tubes from all patients within 24 h of admission. The tubes were inverted gently several times to ensure thorough mixing of the blood and anticoagulant. The samples were then centrifuged at 3500 rpm for 10 min at an ambient temperature of approximately 22 °C, and the supernatants were collected into fresh tubes for aliquoting. The tubes were not inverted or agitated after centrifugation. Samples were stored at 2–8 °C and analyzed within 8 h. The concentrations of all target biomarkers (Aβ1-42, Aβ1-40, p-Tau 181, p-Tau 217, NFL, and GFAP) were quantified using a fully automated chemiluminescent immunoassay analyzer (Shine i2910, Shenzhen Yingkai Biotechnology Co., Ltd., China). The assay is based on a sandwich-type chemiluminescent immunoassay principle. Briefly, plasma samples were incubated with magnetic beads coated with specific capture antibodies. After incubation, an acridinium ester-labeled detection antibody, targeting a distinct epitope on the same antigen, was added to form an immunocomplex and, following a wash step, a trigger solution was introduced to initiate the chemiluminescent reaction. The resultant signal, measured in relative light units (RLUs), is directly proportional to the analyte concentration. The instrument was calibrated prior to sample analysis. All procedures were conducted by qualified technical personnel who remained blinded to the subject and sample information.

### 2.3 Participant data collection

Data were obtained from the patients on admission. The information included demographic and clinical data, as well as information on hematological parameters. The demographic data included sex, age, body mass index (BMI), and years of education. The clinical information comprised the patient’s medical history (hypertension, diabetes, cardiac disease), National Institute of Health Stroke Scale (NIHSS) score on admission, Modified Rankin Scale (MRS) score on admission, MOCA score on admission (baseline level), and location of the stroke. The NIHSS score was used for assessment of neurological deficits in the patients (Adams et al., 1999), with higher scores indicating more severe neurological impairment. Clinical symptoms and daily living activities were assessed using the MRS (Saver et al., 2021), with scores ranging from 0 to 6. Higher scores indicate greater functional disability and poorer ability to perform daily activities. Hematological indicators included plasma levels of Aβ1-40, Aβ1-42, GFAP, NFL, p-Tau181, p-Tau217, Aβ42/40, and the p-Tau217/Aβ1-42 ratio on admission.

Participants underwent follow-up assessments 3 months after the stroke. Cognitive function was evaluated by specifically trained neurologists using the Montreal Cognitive Assessment (MOCA), a tool proven effective for screening cognitive impairment and dementia in the Chinese population (Chinese Stroke Association Vascular Cognitive Impairment Subcommittee, 2024). The MOCA scale is generally preferred to the MMSE scale, particularly in the subacute phase following stroke, as it is more sensitive to the detection of mild cognitive impairment (El Husseini et al., 2023; Van Heugten et al., 2015). Based on the findings of earlier research, subjects with MOCA scores <22 were defined as cognitively impaired (PSCI group), while those with scores ≥22 constituted the cognitively normal group (Lees et al., 2014).

### 2.4 Statistical analysis

Data were analyzed using SPSS 27.0 (IBM Corp., Armonk, NY, USA), using univariate analyses. Normally distributed continuous variables were compared using independent-samples *t*-tests, while non-normally distributed variables were assessed with Mann–Whitney U tests. Categorical variables were compared using chi-square tests. Variables that showed significance (*p* < 0.05) in the univariate analysis were incorporated as independent variables into a multivariate binary logistic regression analysis to identify independent risk factors for PSCI.

Receiver operating characteristic (ROC) curves, with calculation of the area under the curve (AUC), were used to evaluate the predictive efficacy of plasma biomarkers for PSCI. First, ROC analyses were conducted using plasma GFAP and NFL as predictive variables. Subsequently, a predictive model based on the combination of GFAP and NFL was constructed based on the results of binary logistic regression. The model generated a predictive probability value representing the combined predictive indicator, which was calculated as follows: *p* = 1/(1 + e^(−z)), where z = *β*₀ + β₁ × [GFAP] + β₂ × [NFL], where β₀ is a constant and β₁ and β₂ represent the partial regression coefficients (Muller and MacLehose, 2014). Finally, ROC curves for GFAP, NFL, and the combined predictive probability were plotted and compared. The optimal cut-off values for each predictive indicator were determined using the Youden index, and the corresponding sensitivity and specificity values were calculated. *p*-values < 0.05 were considered statistically significant in all statistical analyses.

## 3 Results

### 3.1 Baseline characteristics of the study participants

**Figure 1:**
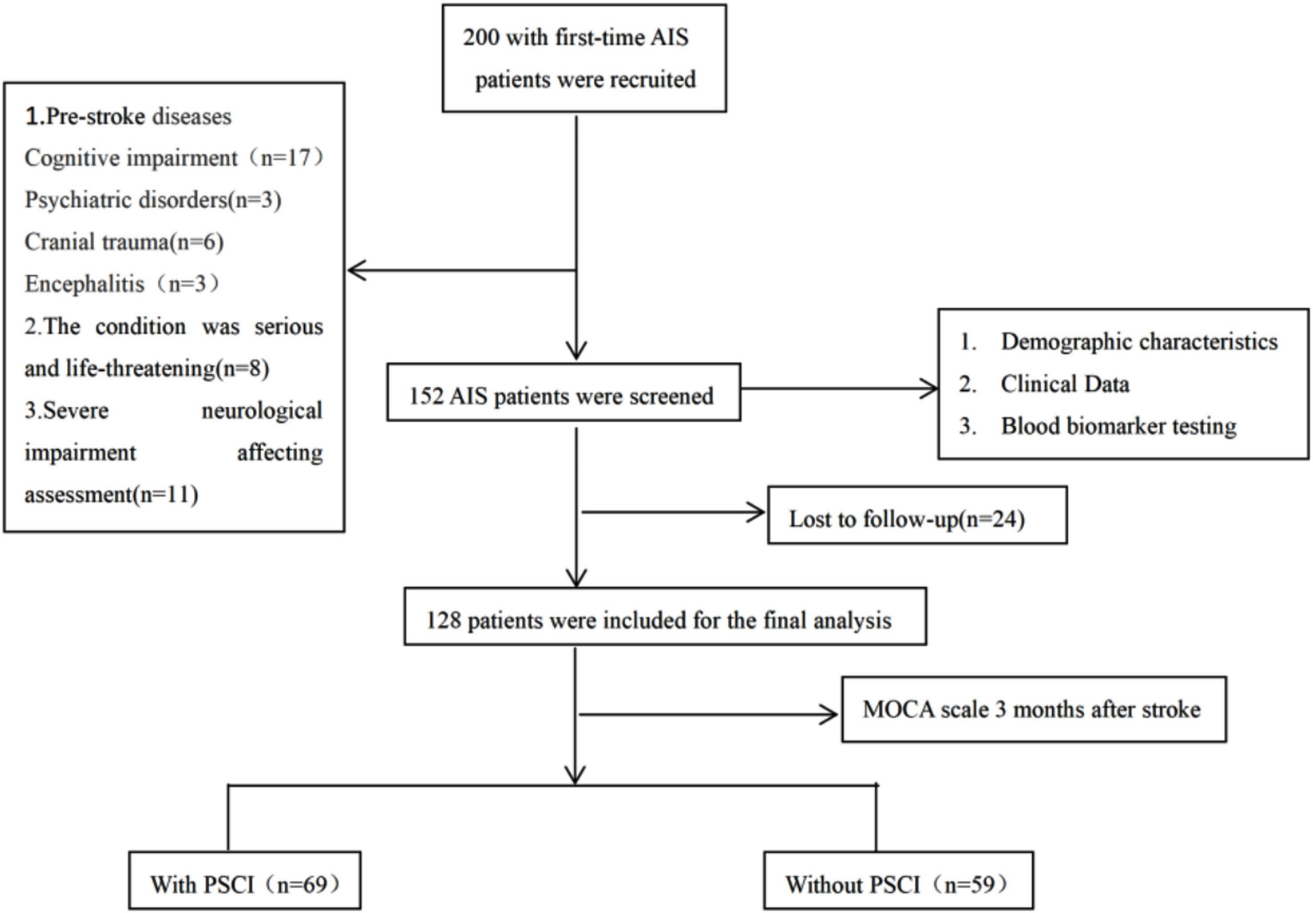
Flowchart of the participant selection process.

Two hundred patients with first-time acute ischemic strokes were initially recruited. After application of the exclusion criteria, 152 participants were selected. Twenty-four individuals were lost to follow-up by the three-month post-stroke follow-up appointment, resulting in the enrollment of 128 patients in the final analysis. Figure 1 presents the study flow diagram. The cohort comprised 92 males (71.9%) and 36 females (28.1%). Age range: 51–80 years, median age 65 years (interquartile range [IQR]: 56.25–71 years). Based on MOCA scores 3 months post-stroke, 128 patients were categorized into the PSCI group and the cognitively normal group. The PSCI group comprised 69 individuals, including 53 males (76.8%) and 16 females (23.2%); the cognitively normal group comprised 59 individuals, including 39 males (66.1%) and 20 females (33.9%). The median plasma GFAP level in the PSCI group was 237.05 pg./mL (IQR: 133.40–597.12 pg./mL), while that in the normal group was 94.16 pg./mL (IQR: 50.07–202.38 pg./mL). The median plasma NFL level in the PSCI group was 146.48 pg./mL (IQR: 117.08–304.70 pg./mL), with a value of 67.06 pg./mL (IQR: 45.72–114.68 pg./mL) in the cognitively normal group.

### 3.2 Univariate and multivariate analyses

Table 1 shows the results of the univariate analysis. It was found that age, prior history of hypertension, NIHSS score on admission, MRS score on admission, plasma GFAP level on admission, and plasma NFL level on admission were significant risk factors for post-stroke cognitive impairment. The results of the multivariate logistic analysis (Table 2) indicate that, after adjusting for age, history of hypertension, NIHSS score at admission, and MRS score, elevated plasma GFAP levels (OR = 1.0027, 95% CI = 1.0002–1.0053, *p* = 0.038) and elevated plasma NFL levels (OR = 1.0046, 95% CI = 1.0006–1.0086, *p* = 0.025) remained independent risk factors for post-ischaemic stroke cognitive impairment. It should be noted that, as GFAP and NFL are measured in pg./mL, their OR values represent a change in risk per 1 pg./mL increase. Therefore, values close to 1.00 are expected and do not imply weak clinical significance. Given that the actual variation in plasma GFAP and NFL levels between patients can reach tens or hundreds of pg./mL, their cumulative effect may have substantially greater clinical relevance.

**Table 1:** Analysis of risk factors for PSCI.

Variable	With PSCI, *n* = 69	Without PSCI, *n* = 59	*p*-value
Demographic characteristics			
Male, %	53 (76.8%)	39 (66.1%)	0.179
Age, year	66 (61, 72.5)	62 (52,70)	0.015
BMI, kg/m^2^	25.03 ± 3.16	24.85 ± 3.98	0.061
Years of education	9 (6, 9)	9 (6, 9)	0.971
Medical history			
Hypertension, *n* (%)	53 (76.8%)	35 (59.3%)	0.033
Diabetes, *n* (%)	20 (29.0%)	16 (27.1%)	0.179
Heart disease, *n* (%)	9 (13.0%)	5 (8.5%)	0.409
Clinical data			
NIHSS score on admission	4 (1, 6)	2 (0, 4)	<0.001
MRS score on admission	2 (1, 3)	1 (1, 2)	<0.001
Site of stroke			0.200
Left cerebral hemisphere, %	31 (44.9%)	21 (35.6%)	
Right cerebral hemisphere, %	15 (21.7%)	21 (35.6%)	
Brainstem or cerebellum, %	14 (20.3%)	13 (22.0%)	
Bilateral multiple lesions, %	9 (13.0%)	4 (6.8%)	
Laboratory data			
Aβ1-40, pg./mL	79.00 (31.46, 110.97)	77.39 (41.64, 100.00)	0.539
Aβ1-42, pg./mL	3.87 (1.53, 6.37)	4.05 (2.46, 5.59)	0.918
Aβ42/40, %	0.06 (0.04, 0.06)	0.06 (0.05, 0.06)	0.193
GFAP, pg./mL	237.05 (133.40, 597.12)	94.16 (50.07, 202.38)	<0.001
NFL, pg./mL	146.48 (117.08, 304.70)	67.06 (45.72, 114.68)	<0.001
p-Tau 181, pg./mL	1.45 (0.78, 2.34)	1.50 (0.69, 2.21)	0.665
p-Tau 217, pg./mL	1.15 (0.63, 2.15)	1.13 (0.51, 2.62)	0.833
p-Tau 217/Aβ1-42, %	0.33 (0.17, 0.83)	0.30 (0.19, 0.66)	0.828

**Table 2:** Results of the multivariate logistic regression analysis.

Variable	OR	95%CI	*p*-value
GFAP	1.0027	1.0002–1.0053	0.038
NFL	1.0046	1.0006–1.0086	0.025
Age	1.0352	0.9944–1.0777	0.092
Hypertension	2.4375	0.8877–6.6928	0.084
NIHSS at admission	1.1671	0.9235–1.4749	0.196
MRS at admission	1.4272	0.7965–2.5575	0.232

### 3.3 Efficacy of plasma GFAP and NFL levels in predicting PSCI

**Figure 2:**
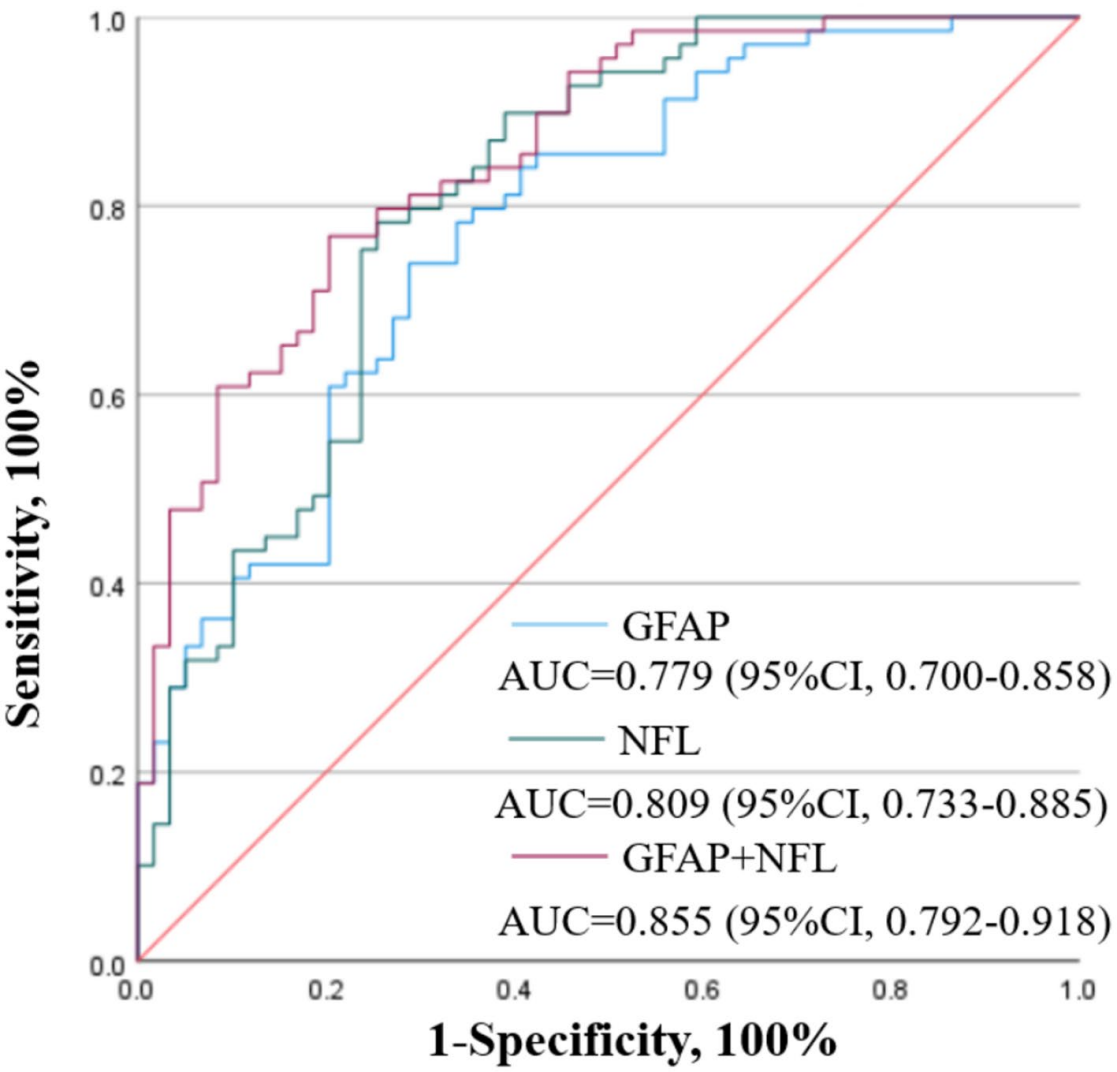
ROC curves for PSCI prediction.

ROC curves were also used to assess the predictive efficacy of plasma GFAP and NFL levels in predicting PSCI (Figure 2). Results demonstrated that the AUC for plasma GFAP alone was 0.779 (95% CI = 0.700–0.858, *p* < 0.001), with an optimal cut-off value of 155.422 pg./mL yielding corresponding sensitivities and specificities of 73.9 and 71.2%. Plasma NFL alone demonstrated superior predictive performance, with an AUC of 0.809 (95% CI = 0.733–0.885, *p* < 0.001) and an optimal cut-off value of 111.437 pg./mL, yielding corresponding sensitivities and specificities of 78.3 and 74.6%. A combined predictive model based on GFAP and NFL was then constructed using binary logistic regression. The AUC of this combined model was improved to 0.855 (95% CI = 0.792–0.918, *p* < 0.001), significantly outperforming that of either single indicator. The optimal cutoff value for determining predictive probability based on the Youden index was 0.513, yielding a sensitivity of 76.8% and a specificity of 79.7% for the model.

In summary, both plasma GFAP and NFL are effective biomarkers for the prediction of PSCI. Furthermore, the predictive model based on the combination of plasma GFAP and NFL levels demonstrated superior predictive efficacy for PSCI.

## 4 Discussion

In recent years, research on biomarkers has emerged as a focal point for studies on neurodegenerative and neuroimmunological disorders (Cummings et al., 2025; Li et al., 2025; Min et al., 2025). Compared to the use of cerebrospinal fluid and PET imaging, blood-based biomarkers are simpler to determine, minimally invasive, and cost-effective (Hansson et al., 2023). These advantages contribute to both increased patient acceptability and easier implementation on a broader scale (Palmqvist et al., 2025). This study employed chemiluminescence assays to prospectively measure the levels of eight plasma biomarkers (Aβ1-40, Aβ1-42, GFAP, NFL, p-Tau181, p-Tau217, Aβ42/40, and p-Tau217/Aβ1-42) in patients with acute ischaemic stroke. This comprehensive assessment aimed to explore the associations between plasma biomarkers and PSCI. The results of the univariate analysis revealed that plasma NFL, plasma GFAP, age, history of hypertension, NIHSS score on admission, and MRS score on admission were risk factors for PSCI. After adjusting for confounding factors including age, history of hypertension, NIHSS score at admission, and MRS score at admission, it was found that plasma GFAP and NFL levels were independent risk factors for PSCI development. To compare these results with previous research findings, Wang et al. (2021) reported that plasma NFL was an independent risk factor for PSCI, consistent with the present results. In contrast to the current findings, Huang et al. (2022) have suggested that plasma p-tau181 may be a potential biomarker for PSCI. However, Li et al. (2025) have shown that although tau protein is an effective biomarker for Alzheimer’s disease (AD), there is limited evidence supporting its clinical application in PSCI. Although *β*-amyloid is a key biomarker for AD, it was not found to be a predictor of PSCI in this study. However, previous studies have focused predominantly on individual markers, such as tau protein, β-amyloid (Aβ), GFAP, and NFL. Our research indicates that elevated plasma levels of GFAP and NFL at admission in patients with acute ischemic stroke are associated with an increased risk of permanent stroke-related disability. ROC curve analysis demonstrated that both plasma GFAP and NFL were effective in predicting the development of PSCI. The present study further revealed that, compared to either plasma GFAP or NFL alone, the GFAP-NFL combination was more effective in predicting PSCI.

The onset and progression of PSCI represent a multifactorial pathological process involving complex mechanisms. Despite extensive research both domestically and internationally, the precise pathophysiological mechanisms underlying PSCI remain unclear. Research suggests that these mechanisms may be associated with factors such as cerebral hypoperfusion, disruption of the blood–brain barrier, white matter degeneration, and neuroinflammation (Coenen et al., 2024; Wang et al., 2022). The blood–brain barrier serves as the brain’s primary defensive barrier. When compromised, inflammatory mediators and circulating toxins may enter the brain, causing axonal damage and neuronal degeneration, ultimately resulting in cognitive impairment (Nation et al., 2019; Rajeev et al., 2022).

Neurofilaments are the primary component of the axonal cytoskeleton, where they are responsible for maintaining the structural integrity of neuronal cells (Jung and Damoiseaux, 2024). NFL is a neurofilament subunit. Axonal injury or neuronal death induces calpain-mediated proteolytic cleavage of neurofilaments, resulting in the release of the NFL fragment into the cerebrospinal fluid and blood (Kahn et al., 2025). This activates microglia and macrophages, thereby initiating an inflammatory response. NFL-induced microglial activation may form a feedback loop, exacerbating processes associated with neurodegeneration. NFL is regarded as a marker of axonal injury and neurodegeneration (Sewell et al., 2025). Numerous studies have indicated the efficacy of NFL as a biomarker in various neurodegenerative diseases, including Alzheimer’s disease (AD), Parkinson’s disease (PD), multiple sclerosis (MS), and amyotrophic lateral sclerosis (ALS) (Aamodt et al., 2021; Krogseth et al., 2023; Kuhle et al., 2019). Previous research has indicated that elevated levels of NFL are associated with poorer cognitive function (Ramani et al., 2021). As peripheral blood sampling is simpler and non-invasive compared to the collection of cerebrospinal fluid, research into blood biomarkers is particularly important. However, the relationship between plasma NFL and PSCI is not fully explained. This study revealed an association between elevated plasma NFL levels and an increased risk of cognitive impairment following ischemic stroke. This suggests that plasma NFL may serve as a potential biological marker for PSCI, with good predictive efficacy.

Astrocytes, the most abundant glial cells in the central nervous system, contain a cytoskeleton composed of numerous microtubules that are involved in a variety of physiological processes (Leipp et al., 2024). It has been shown that GFAP, a key protein in the glial cytoskeleton, is crucial for the maintenance of neuronal mechanical strength and the integrity of the blood–brain barrier (Park et al., 2024). Damage or death of astrocytes due to neuroinflammation leads to the release of GFAP into the cerebrospinal fluid and blood (Hase et al., 2018). Studies have indicated the presence of elevated GFAP levels in the cerebrospinal fluid and blood in various neurodegenerative diseases and brain injuries (Andres Benito et al., 2018; Tang et al., 2023; Zou et al., 2024), including AD, PD, dementia with Lewy bodies, frontotemporal dementia, and Creutzfeldt-Jakob disease. The present study demonstrates an association between plasma GFAP levels and cognitive impairment following ischaemic stroke. However, it is noteworthy that previous studies have demonstrated that plasma GFAP and NFL are associated not only with central nervous system diseases but also with peripheral neuropathies, such as diabetic polyneuropathy (DPN) (Nasr-Eldin et al., 2024) and Charcot–Marie–Tooth disease (CMT) (Pretkalnina et al., 2024).

However, the study has several limitations. First, the study was conducted in a single institution, using a relatively small patient cohort. Differences in testing methods and performance may exist between different institutions, thus restricting the generalizability of the findings. Secondly, this study employed a cross-sectional design to examine the associations and predictive efficacies of plasma biomarkers for PSCI during the acute phase of ischemic stroke (upon admission). Consequently, the levels of these plasma biomarkers were not re-assessed at 3 months post-stroke or thereafter, and the study was therefore unable to explore the relationship between longitudinal alterations in biomarker levels and changes in cognitive function. Thirdly, follow-up cognitive assessments were undertaken only at the three-month follow-up time point, and follow-up was not extended for 6 months post-stroke or beyond.

In summary, this study identified that elevated plasma GFAP and NFL levels are associated with an increased risk of post-ischemic stroke cognitive impairment. The combination of the two parameters showed superior predictive efficacy for PSCI. Plasma GFAP and NFL may represent potential biological markers for PSCI.

## Data Availability

The original contributions presented in the study are included in the article/supplementary material, further inquiries can be directed to the corresponding author.
